# Human Skin Drug Metabolism: Relationships between Methyl Salicylate Metabolism and Esterase Activities in IVPT Skin Membranes

**DOI:** 10.3390/metabo13080934

**Published:** 2023-08-09

**Authors:** Krishna C. Telaprolu, Jeffrey E. Grice, Yousuf H. Mohammed, Michael S. Roberts

**Affiliations:** 1 Therapeutics Research Centre, Frazer Institute, The University of Queensland, Woolloongabba, QLD 4102, Australia; krishna.chaitanyatelaprolu@certara.com (K.C.T.); jeff.grice@uq.edu.au (J.E.G.); 2 School of Pharmacy, The University of Queensland, Woolloongabba, QLD 4102, Australia; 3 UniSA—Clinical and Health Sciences, University of South Australia, Adelaide, SA 5000, Australia; 4 Therapeutics Research Centre, Basil Hetzel Institute for Translational Medical Research, The Queen Elizabeth Hospital, Woodville, SA 5011, Australia

**Keywords:** esterase distribution, human skin membranes, ester drugs, methyl salicylate, dispase II, skin metabolism, epidermal metabolism, dermal metabolism

## Abstract

The presence of esterase enzymes in human skin and their role in drug metabolism has been reported, but their distribution in the various skin layers and the relative contributions of those layers to metabolism is poorly defined. To gain further insight into esterase distribution, we performed in vitro skin permeation of a commercial 28.3% methyl salicylate (MeSA) cream (Metsal™) in Franz diffusion cells, using a range of human skin membranes, all from the same donor. The membranes were viable epidermis separated by a dispase II enzymatic method, heat separated epidermis, dermatomed skin, and dermis separated by a dispase II enzymatic method. Methyl salicylate and its metabolite, salicylic acid (SA), were measured by high-performance liquid chromatography. Alpha naphthyl acetate and Hematoxylin and Eosin staining provided qualitative estimations of esterase distribution in these membranes. The permeation of methyl salicylate after 24 h was similar across all membranes. Salicylic acid formation and permeation were found to be similar in dermatomed skin and dermis, suggesting dermal esterase activity. These results were supported by the staining studies, which showed strong esterase activity in the dermal–epidermal junction region of the dermis. In contrast with high staining of esterase activity in the stratum corneum and viable epidermis, minimal stained and functional esterase activity was found in heat-separated and dispase II-prepared epidermal membranes. The results are consistent with dispase II digesting hemidesmosomes, penetrating the epidermis, and affecting epidermal esterases but not those in the dermis. Accordingly, whilst the resulting dispase II-generated dermal membranes may be used for in vitro permeation tests (IVPT) involving esterase-based metabolic studies, the dispase II-generated epidermal membranes are not suitable for this purpose.

## 1. Introduction

Various skin membranes are used in in vitro permeation test (IVPT) studies to assess the skin absorption of drugs from topically applied products. The studies are then often used to predict in vivo absorption of drugs into their viable epidermal and dermal sites of action, as it is difficult to determine the time course of drugs in the viable epidermis and dermis under in vivo conditions [1,2]. However, any underlying in vitro–in vivo relationship is dependent on both the physical barrier properties of the stratum corneum and other layers, as well as the impact of metabolism in those layers. Any difference in the metabolism of topical drugs in these layers between ex vivo skin membranes and in vivo may adversely impact the useability of IVPT-in vivo skin permeation relationships [3]. Human skin contains phase-I cytochrome P450 enzyme families, esterases, dehydrogenases, along with phase II enzymes [4,5,6]. Apart from acting as a barrier to permeation, the skin is capable of detoxifying xenobiotics that penetrate the skin by bio-transforming them into less harmful metabolites. In addition, it can activate penetrating substances enzymatically by converting prodrugs to their active forms, catalyzed by specific metabolic enzymes present in the skin [7]. Understanding skin metabolism has become increasingly relevant in the design of prodrugs to target a specific site of action [8] in a drug discovery setting and safety assessment of consumers and drug products [9]. The activity of human skin enzymes has been described in various published reviews [9,10]. 

Among various enzymes in the skin, esterases that are reported to be located in the endoplasmic reticulum and cytosol of mammalian tissues [11] have been exploited to convert a number of substances, such as prodrugs, to their active forms. These crucial esterases have a major influence on the efficacy, safety, and skin penetration of various drugs, including naltrexone ester prodrugs [12], ethyl nicotinate (to nicotinic acid) [13], and glucocorticoids such as betamethasone 17-valerate, prednisolone diester prednicarbate [9], and tazarotene [14]. Esterase-catalyzed biotransformation of drugs has been demonstrated in human skin homogenates with fluorescein acetate, prednicarbate [15], penta-ethyl ester prodrug of DTPA [16], and in viable full-thickness human and pig skin with retinyl palmitate conversion to retinol [17]. The metabolic conversion of applied actives by esterases has been evaluated in full-thickness skin in several in vitro permeation studies reported in the literature, but the location of responsible enzymes and the contribution of those enzymes from each layer has not been assessed in human skin [18,19,20]. Lau et al. [11] reported that the stained esterase activity in porcine ear skin is mainly localised in the stratum corneum and viable epidermis, with much less in the dermis and is substantially reduced by frozen storage and even more so by epidermal heat separation. They showed that the relative hydrolysis of aspirin to salicylate in freshly prepared skin was significantly greater than in frozen, heat-separated, or vehicle-treated and control skin. Roberts et al. studied salicylate formation from topically applied methyl salicylate formulations in humans in vivo by performing urine analysis [21] and by clinical microdialysis [3]. The latter study also showed that the human IVPT metabolism of methyl salicylate using a heat-separated epidermal membrane was much lower than found in in vivo using dermal micro dialysate [3]. However, a limitation of this analysis is that clinical microdialysis sampling occurs in the reticular dermis, usually up to 1 mm below the surface of the skin. Accordingly, this metabolism reflects the accumulated stratum corneum, viable epidermal, superficial dermal, and mid-dermal metabolism. 

**Figure 1:**
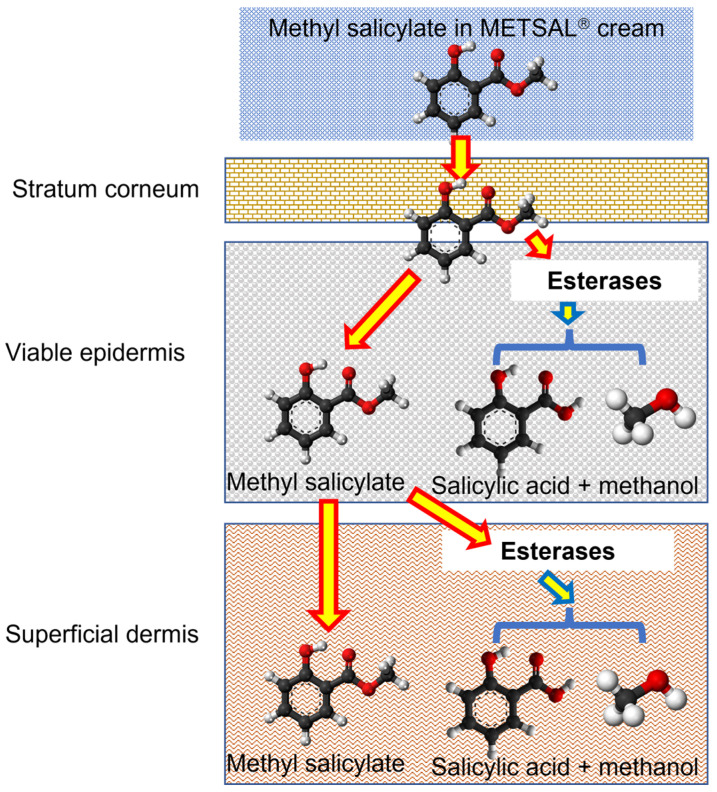
Schematic representation of methyl salicylate metabolism by skin esterases to salicylic acid by breaking the ester chemical bond in the molecule [24,25,26].

In this work, we sought to better understand the spatial human skin metabolism of methyl salicylate in human skin. Methyl salicylate was chosen for this work as it is a commonly available over-the-counter topical agent used for pain and inflammatory conditions affecting muscles and joints and converted to salicylic acid and methanol by the skin carboxyesterases (Figure 1) [22]. In recognizing the impaired esterase metabolism of heat-separated human epidermal membranes now widely used in human IVPT studies, we prepared a new human epidermal membrane separated from the dermis using the dispase II enzyme. This membrane was then mounted and analyzed using an in vitro permeation test (IVPT) method using a topically applied commercial methyl salicylate (Metsal™) cream, and the results compared to IVPT studies using dermatomed fresh ex vivo skin, dermis remaining after viable epidermis separation and non-viable epidermis obtained by heat-separation of full-thickness skin [23]. We then sought to relate our findings with stained esterase activity. To pinpoint the distribution of esterases in the different membranes, they were stained with a non-specific esterase staining dye, α-naphthyl acetate, to understand the distribution of esterase enzymes and relate that with skin permeation results.

## 2. Materials and Methods

### 2.1. Chemicals

Metsal^™^ cream (iNova Pharmaceuticals, Chatswood, Australia) containing 28.3% methyl salicylate was used in the present study. Sodium azide, phosphate buffered saline sachets (pH 7.4), phosphoric acid of analytical grade, methyl salicylate, and salicylic acid, Fast Blue RR salt, α-naphthyl acetate, Mayer’s hematoxylin, ethylene glycol butyl ether, and Dispase II were purchased from Sigma–Aldrich (Castle Hill, Australia). Tris(hydroxymethyl)aminomethane (TRIS) was purchased from Merck (Darmstadt, Germany), acetone was purchased from Chem Supply (Gillman, Australia), methanol and acetonitrile were purchased from VWR Chemicals (Campbellfield, Australia). Tissue Tek Optimum Cutting Temperature (OCT) embedding medium, cryomold^®,^ was obtained from ProSciTech (Thuringowa, Australia). All solution preparations used Milli-Q water when required. Croda Australia kindly supplied Brij™ O20/Volpo N20.

### 2.2. Membrane Preparation

**Figure 2:**
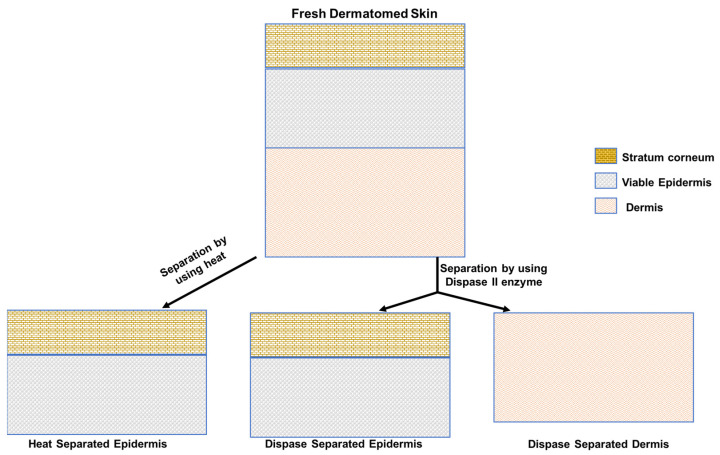
Schematic representation of methods used for preparation of various membranes used in the IVPT study of Metsal™ cream.

Human skin membranes were prepared from freshly excised skin and provided with informed consent by donors undergoing elective abdominoplasty. All three donor skins used in these studies were from female individuals aged between 45 to 55 years. The acquisition and use of the donated skin were approved by the Metro South Hospitals and the University of Queensland Human Research Ethics Committees (Approval number: 2008001342). The subcutaneous fat was removed, and the skin was dermatomed at 500 µm thickness using an Integra^®^ Padgett^®^ air dermatome (Model C Padgett instruments, Inc., Princeton, NJ, USA). Epidermal membranes were prepared by heat separation and enzymatic separation by the dispase II enzyme. Enzymatic epidermal separation was carried out by placing the dermatomed skin of approximately 500 µm on a sterile gauze imbibed with dispase II enzyme solution of 7 mL (2.4 IU/mL in HEPES buffer pH 7.4) and incubated at 37 °C in a water bath for 90 min. The dermis membrane that remained after enzymatic separation was also used for the permeation study. Figure 2 shows the schematic representation of preparation of various membranes described here.

### 2.3. In Vitro Skin Permeation of Methyl Salicylate from Metsal™ Cream

The prepared skin membranes were mounted separately on Franz-type diffusion cells. Dermatomed skin, heat-separated epidermis, and dispase-separated epidermis were placed with the stratum corneum (SC) facing upwards toward the donor chamber, while the dermis was mounted with the dermal–epidermal junction facing upwards. The exposed skin surface area was 1.33 cm^2^. Three replicates each from 3 different skin donors of the four membranes (dispase-separated epidermis, heat-separated epidermis, dermatomed skin, and dermis) were used, and for non-specific esterase staining, four membranes from single donor data were used in this work. 

After mounting the skin, both donor and receptor compartments were filled with PBS (0.05% sodium azide), and the cells were equilibrated in a water bath for 30 min at 35 ± 1 °C with continuous stirring (magnetic stirrer bars) to maintain a skin surface temperature of 32 °C. The resistance of the membranes was measured using a digital multimeter. Apart from the dermis, membranes with a resistance lower than 20 kΩ were discarded for the study. Both the compartments were then emptied, and the excess liquid on the membrane surface was dabbed with KimWipes^™^ from Kimberly-Clark^™^Brisbane, Australia. The receptor compartment was filled with freshly prepared 6% Brij™ O20 in PBS to enhance the solubility of permeating actives in the receptor fluid. The Metsal^™^ cream was applied with a positive displacement pipette and spread with a syringe plunger to achieve a dose of 0.1 g/cm^2^. Receptor samples (200 µL) were collected at 0.5, 1, 2, 4, 6, 8, 12, 18, 24 h, and a pre-warmed receptor medium was used to replace it. 

### 2.4. Sample Analysis 

The receptor samples were analysed by a validated HPLC method using a Shimadzu Prominence Ultra flow liquid chromatography (UFLC) system with a SIL20-AHT autosampler and an SPD20A UV detector. The samples were prepared by mixing 50 µL of the sample aliquotes with 50 µL each of 25% ethanol in water and propylparaben 25 µg/mL in ethanol (as internal standard). The standards were prepared by mixing 50 µL of standard with 50 µL each of propylparaben 25 µg/mL of ethanol and 6% (*w/v*) Brij™ O20 in PBS. The column used was Luna C18-2, 150 × 4.6 mm, 5 µm. The mobile phase was 52:48 Acetonitrile: 0.1% phosphoric acid in water at a flow rate of 1 mL/min, and the injection volume was 50 µL [27]. Salicylic acid, methyl salicylate, and propylparaben were detected at 235 nm and had retention times of 3.5, 5.5, and 7.5 min, respectively. The method provided good precision and linearity in the required concentration range (methyl salicylate; 1.56–500 μg/mL, R^2^ = 0.999: salicylic acid; 0.78–500 μg/mL, R^2^ = 0.999). The lower limit of quantification (LLOQ) for methyl salicylate and salicylic acid was 0.2 μg/mL. The chromatography software used was LC solutions^®^ (Shimadzu, Kyoto, Japan).

### 2.5. Non-Specific Esterase Staining by α-Naphthyl Acetate 

The four test membranes (dispase separated epidermis, heat separated epidermis, dermatomed skin/frozen full thickness skin, and dermis remained after both epidermis separation) were cut into 4 mm^2^ pieces and embedded in OCT compound for cryosectioning. Cryotome sections of 5 µm thickness (Leica CM1850 cryomicrotome (Leica, Bensheim, Germany) were mounted on positively charged glass slides. Sections were treated with α-naphthyl acetate to stain all the non-specific esterase enzymes. The sections on slides were fixed with citrate buffer: acetone: methanol solution for 1 min, then rinsed thoroughly with deionised water. The α-naphthyl acetate solution (10 mg/mL) was prepared using ethylene glycol butyl ether. The prepared solution was added to TRIS buffer (pH 7.5) containing Fast Blue RR salt. The slides were incubated in α-naphthyl acetate solution for 15 min at 37 °C. After incubation, the slides were washed in deionized water and counterstained by placing them in Mayer’s hematoxylin for 5 min, followed by washing in tap water. The developed images were captured using an Olympus Slide scanner VS120 (rm4026) optical microscopy and reviewed with OlyVIA software version 2.9.1.

### 2.6. Data and Statistical Analysis

The amounts (µg/cm^2^) of methyl salicylate and salicylic acid permeated at the end of the study (24 h) were converted to percentages of the applied dose of methyl salicylate applied to each membrane. These percentages, as well as the percentage of total actives, permeated (MeSA + SA) through each membrane, were analyzed by one-way ANOVA with post-hoc comparisons by Tukey’s test, using GraphPad Prism version 8.2.0 (GraphPad Software Inc., La Jolla, CA, USA)

## 3. Results

### 3.1. In Vitro Skin Permeation Testing (IVPT) of Metsal™ Cream 

**Figure 3:**
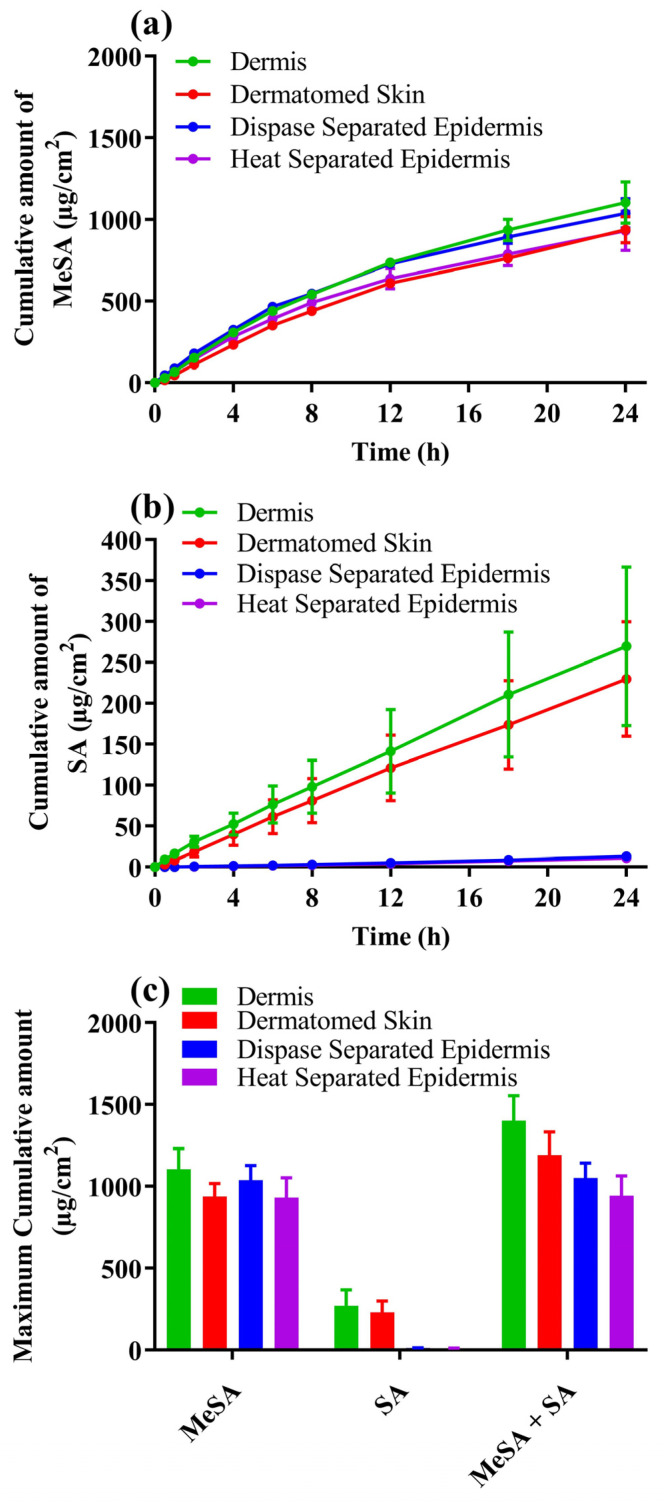
In vitro permeation profiles of Metsal™ Cream applied on different skin membranes with 6% Brij™ O20 as receptor phase: (**a**) Cumulative amount versus time (Mean ± SEM *n* = 3) of methyl salicylate; (**b**) Cumulative amount versus time (Mean ± SEM *n* = 3) of salicylic acid; (**c**) Total amount of the actives permeated (Mean ± SEM *n* = 3).

Figure 3a,b show the cumulative amount versus time profiles for methyl salicylate permeation, salicylic acid formed, and permeated, respectively, across various membranes obtained from the same donor. Table 1 shows the percentages of the applied dose permeated as methyl salicylate, salicylic acid, total actives, and SA to MeSA ratio from the various membranes. We found no significant difference between the permeation profiles of methyl salicylate (Figure 3a and Table 1) from the different types of membranes used. There was no significant difference in the percentage of applied dose permeated as the total amount of actives (methyl salicylate + salicylic acid) in any of the membranes (Figure 3c and Table 1). As illustrated in Figure 3b, the cumulative amount of salicylic acid formed and permeated was similar in dermatomed skin and dermis (Figure 3b) but very low in dispase-separated epidermis and heat-separated epidermis. Results in Table 1 show that SA to MeSA formation and the permeation ratio from the dispase-separated epidermis and heat-separated epidermis was significantly different when compared to dermis membrane but similar between dermatomed and dermis membranes. 

### 3.2. Non-Specific Esterase Staining by α-Napthyl Acetate 

**Figure 4:**
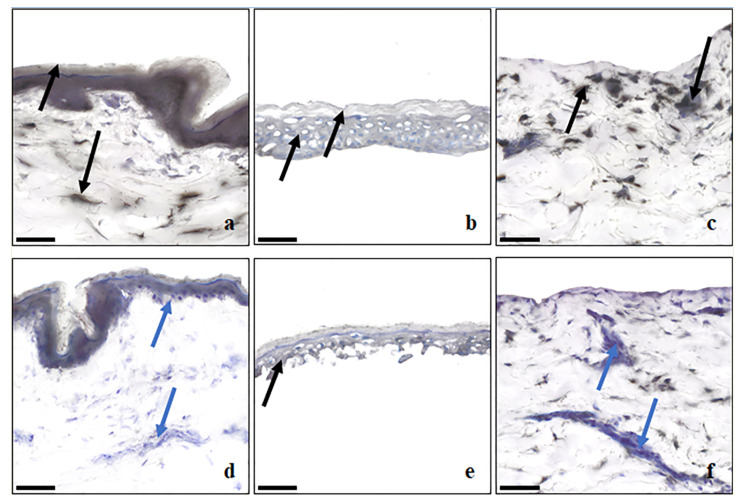
Distribution of esterase activity in the cryosections of human abdominal skin visualized by nonspecific esterase enzyme staining (**a**) dermatomed skin; (**b**) dispase separated epidermis; (**c**) dermis remained after epidermis separated by dispase enzyme; (**d**) full thickness skin, frozen for three months; (**e**) heat separated epidermis; (**f**) dermis remained after epidermis separation by heat. Black to grey color indicates the presence of esterase distribution, represented by black arrows, and the blue to violet color from the counter staining by hematoxylin represents the absence of esterases in that area, represented by blue arrows. Scale bar is 50 µm.

The esterase enzymes in the skin sections of the different membranes were stained with α-naphthyl acetate, followed by counterstaining with Mayer’s hematoxylin. Skin esterases convert α-naphthyl acetate to α-naphthol, which binds with the diazonium ions in the staining solution to produce black-to-grey staining of the skin areas where esterases are distributed. The staining intensity depends on the length of incubation and the population density of esterases present [11]. Counterstaining with hematoxylin was used to differentiate layers of the skin based on morphology, as well as to reveal cellular regions lacking esterase distribution, indicated by a blue-violet stain. A description of the location of the enzymes in each layer is given below in Table 2. All SC membrane layers were stained black, indicating the presence of esterase activity on the skin surface. The intensity of staining was greater in dermatomed skin (Figure 4a) than in epidermal membranes from the same donor. The dermis of the same donor demonstrated strong esterase activity in different smaller localized regions and near the dermal–epidermal junction (Figure 4c). The intensity of staining in the dermis (Figure 4c) obtained from enzymatic epidermis separation was also similar to dermatomed skin dermis, supporting the observations of similar amounts of salicylic acid from both dermatomed skin and dermis. The dispase-separated epidermis (Figure 4b) showed more uniform esterase staining than the heat-separated epidermis (Figure 4e) from the same donor. The staining of frozen full-thickness skin (Figure 4d) and heat-separated membranes was markedly reduced (Figure 4e,f), indicating that esterase activity was reduced upon freezing or heating of the skin membranes. In addition, Bajza et al. have also shown a reduction of P-glycoprotein function upon skin freezing [28].

## 4. Discussion

The role of skin as a metabolizing and detoxifying barrier has gained renewed interest in recent years. Prodrugs are routinely used in various dosage forms, including topical products, to improve potency following metabolism. Assessing the penetration of drugs that are metabolized or can potentially be metabolized in the skin requires in vitro models that are capable of demonstrating metabolic activity. This can be of particular relevance when a generic product is tested for bioequivalence with a reference-listed product. Traditional preclinical tools, such as the in vitro permeation test with heat-separated epidermis, are limited in their ability to provide information about drug metabolism and may, therefore, provide false estimates of bioequivalence. This drawback can be overcome by performing permeation studies with membrane preparations containing different skin layers, in which metabolic enzyme distribution and function are likely to vary. We chose to study ex vivo human skin membranes obtained from human donors rather than artificial skin models such as reconstructed human skin membranes [29,30] and skin-on-chip technologies [31]. These alternatives, while often used to screen topical responses, differ from human skin in their barrier properties and, importantly for our work, are unable to replicate the normal distribution of enzyme activity in the different skin layers. The ex vivo skin membranes chosen for this work were freshly dermatomed skin (comprised of both epidermis and dermis), epidermis only (non-viable heat separated and viable Dispase II enzyme separated), and dermis only. This allowed us to selectively find evidence of the location of functional metabolic enzymes, which was supported by the use of non-specific esterase staining. Esterase distribution in human skin has previously been reported by Montagna [32], who used full-thickness excised skin biopsies from the scalp, back, chest, palm, and axilla of human volunteers. As noted earlier, the spatial distribution of stained esterases in pig skin has been studied by Lau et al. [11], who reported a dominant distribution in the stratum corneum and viable epidermis with much less in the dermis. They also found that frozen storage and epidermal heat separation had significantly less esterase activity than found in fresh skin. The results shown in Figure 3 suggest that the distribution of stained esterases in human skin parallels that in pig skin.

A key unexpected finding in this work was the high esterase activity in the dermis in dermatomed skin and after dispase II separation. As shown in Table 1, Figure 2 and Figure 3, stained and functional activity of dermal esterase appear to be similar in dispase II separated dermis and in dermatomed skin. Our findings for methyl salicylate corroborate the findings of Muller et al. for nicotinate derivatives [33]. However, they contrast with those of Lau et al. [11], who concluded that no functional esterase was present within the porcine dermis based on their staining studies. However, Tokudome et al. [34] have implied with their comment, “esterase specific activity was higher in the epidermis than the dermis (data not shown)”, that some esterase activity does exist in the human dermis.

The present finding of dispase II adversely affecting viable epidermal membranes, but not dermal membranes, was unexpected, as more than 95% viable epidermal cell viability, as measured by the trypan blue dye exclusion, has previously been reported [35]. However, Kitano and Okada also reported an increase in the intercellular spaces of the viable epidermis, and this is consistent with dispase II digestion of viable epidermal hemidesmosomes [36] and penetration, perhaps with some enzyme disruption en route. It is not known why dermal esterase appears to be unaffected by dispase II. Whilst a slow dermal penetration may be anticipated for dispase based on its size (MW 36 kDa), we have reported dermal diffusion of larger solutes [37]. Our earlier studies [3] and those of Lau et al. [11] have suggested that using heat to separate human epidermal membranes greatly diminished the stained and functional activity of the epidermal esterase. Our results in Table 1 are consistent with these findings. Table 1 also suggests that a dispase II separation results in a substantial loss of both stained and functional epidermal esterase activity. In contrast, Tokudome et al. [34] suggested that epidermal and dermal esterase activity and protein content was unaffected by heat separation.

Whilst this work has shown that dispase II has adversely affected viable epidermal esterase metabolism, it is possible that the shorter 0.5% dispase II incubation time of 30 min at 37° and associated mechanical peeling, although performed with mouse skin [38] may have caused less esterase inactivation than the 90 min at 37° and similar 2.4 IU/mL dispase II concentration used here (2.4 IU/mL~0.48% dispase II [39]) with dermatomed human skin. Epidermal cytotoxicity with dispase II is known to be low [40], with our own work and that of others [36] showing good cell viability based on dye exclusion and viable epidermal cellular redox state. Accordingly, further studies are needed to define which viable epidermal functions are preserved using an optimal dispase II separation methodology.

Two limitations of this study are that it has been based on skin collected from female abdominoplasties and focused on skin suitable for use in IVPT. Consequently, potential confounders in skin metabolism arising from gender, age, body site, and skin condition differences are not considered. Further, there is a potential for other fluids (blood) and adipose tissue to also be involved in the overall skin metabolism, although neither is quantified in IVPT studies. A key premise in undertaking IVPT studies is that topical drug action is mainly directed at the stratum corneum, viable epidermis, and superficial dermis. A highly vascularized (blood and lymphatics) system in the superficial dermis is very effective in carrying the bulk of absorbed topically applied drugs directly into the systemic blood [41]. Another important consideration is the treatment of the membrane prior to its use for *IVPT*. Bronaugh et al. showed that conversion to salicylic acid occurred in both freshly excised viable full-thickness guinea pig skin and in non-viable skin [42]. Our work and others have shown that fresh viable skin generally possesses greater enzyme activity than skin that has been through some treatment process, such as frozen storage, epidermal heat separation, or chemical treatment.

## 5. Conclusions

This study has shown the stained and functional spatial distribution of esterase in human skin using methyl salicylate as the probe drug. A key finding is evidence for significant esterase activity in the upper dermis, which can be separated from dermatomed skin using the dispase II separation technology described in this paper. However, it is well known that, whilst enzymes like dispase II completely separate the viable epidermis from the dermis, they can destroy important cell machinery [40]. This work suggests that, whilst dispase II destroys viable epidermal esterases, it does not affect the enzyme activity of the residual dermis. Accordingly, whilst a dermal membrane prepared by the dispase II separation processes described here may be useful for IVPT studies, epidermal membranes retaining the same viable epidermal enzyme activity as found in fresh skin must be prepared by an alternative epidermal separation method. Whilst mechanical or blister suction viable epidermal membranes from full-thickness human skin [40] are possibilities, such membranes are unlikely to meet the required IVPT criteria of being sufficient in size and consistent in permeability for multiple diffusion cells. We conclude that further studies may be warranted on dispase II separated epidermal and dermal membranes, given that it does provide such a high retention of viable epidermal cell viability [36] and dermal esterase activity, as shown here.

## Figures and Tables

**Table 1:** Percentage of dose permeated as methyl salicylate and salicylic acid from various membranes after application of Metsal™ cream. All values are Mean ± SEM (*n* = 3).

Membrane Type	Applied Methyl Salicylate (mg/cm^2^)	% As Methyl Salicylate	% as Salicylic Acid	% as Total Actives	Ratio (SA/MeSA)
Dermis	25.47 ± 1.36	4.44 ± 0.68	1.16 ± 0.31	5.61 ± 0.74	0.26
Dermatomed skin	26.3 ± 0.75	3.59 ± 0.35 ^a^	0.97 ± 0.25	4.56 ± 0.56 ^b^	0.27 ^c^
Dispase-separated epidermis	27.2 ± 1.28	3.88 ± 0.46 ^a^	0.05 ± 0.004 *	3.93 ± 0.47 ^b^	0.013 ^#^
Heat-separated epidermis	28.21 ± 2.23	2.88 ± 0.79 ^a^	0.03 ± 0.01 *	3.86 ± 0.8 ^b^	0.010 ^#^

*: Significantly different when compared to dermis for % dose permeated as salicylic acid. ^a^: Not significantly different when compared to dermis for % dose permeated as Methyl Salicylate. ^b^: Not significantly different when compared to dermis for % dose permeated as both actives combined. ^c^: Not significantly different when compared to dermis for the % dose permeated actives ratio (SA/MeSA). ^#^: Significantly different when compared to dermis for the % dose permeated actives ratio (SA/MeSA).

**Table 2:** Description of location of esterase enzymes in various layers indicated by alpha naphthyl acetic acid staining.

Membrane Type	Location of Esterase Enzymes
Dermatomed Skin	Prescence of esterase staining indicated by black and grey color staining uniform across the epidermal regions and pockets of staining areas across dermis.
Dispase-Separated Epidermis	Prescence of esterase staining indicated by uniform grey color staining across SC and VE parts of the membrane.
Dermis (remained after dispase-separated epidermis)	Prescence of esterase staining indicated by pockets of black color staining distributed throughout dermis and more towards the dermal epidermal junction.
Frozen full thickness skin	Decrease in esterase activity because of freezing indicated by appearance of blue to violet color because of hematoxylin staining in the epidermis regions and more regions in dermis.
Heat-Separated epidermis	Decrease in esterase activity because of heat application indicated by appearance of blue to violet color because of hematoxylin staining in the epidermis regions and more regions in dermis.
Dermis (remained after HSE separation)	Decrease in esterase activity because of heat application indicated by appearance of blue to violet color because of hematoxylin staining throughout dermis and more towards the dermal epidermal junction.

## Data Availability

Not applicable.
